# Removal of a Horse-Shoe Pessary from the Female Bladder

**Published:** 1869-12

**Authors:** W. H. Byford

**Affiliations:** Chicago


ARTICLE XLV.

### REMOVAL OF A HORSE-SHOE PESSARY FROM THE FEMALE BLADDER

BY W. H. BYFORD, M.D., of Chicago.

Reported by B. T. BUCKLEY, MD., Freeport, Ill., President of Stephenson Co. Med. Soc.

On the night of the 28th of February last, was called to visit Mrs B., a young married lady, residing about two miles from the city. Found her suffering severely from pain in the region of the bladder. She informed me that her father's family physician had visited her on that day, and, after an examination, told her that she was laboring under antiversion of the uterus, and that it would be necessary to introduce some instrument to keep the organ in situ. Her physician, being in very poor health, was taken worse at this visit (and it was the last one he ever made), went home, took to his bed, lingered about five weeks, and died. Apprehending that there was some retention of urine, I introduced the catheter, but found the bladder empty, or nearly so. Examined the vagina, but found no pessary. There was slight prolapsus, with anti-flexion. She had become pregnant just previous to this occurrence; and I supposed that these paroxysms of pain were the result of increased sensibility of the uterus and urinary organs, consequent upon partial displacement, and such as we sometimes meet with in cases of recently married women, without any displacement of the uterus whatever. I visited her two or three times a week, for a short time, without any benefit. Drs. DePuy and Mease were called in consultation. After receiving a history of the case, and making an examination, concurred in my opinion or diagnosis of the case. I carried out the suggestions as to treatment, made by the consulting physicians, a short time, without any improvement. The paroxysms became more frequent and severe. By this time, the patient was suffering from constipation, dysuria, and more or less tenesmus. The uterus had passed down considerable below its normal position. I several times attempted to put her under the influence of chloroform, for the purpose of making a more thorough examination, but the effect upon the patient was so distressing, that I was compelled by patient and friends to desist. She gradually grew worse; the symptoms became somewhat alarming from almost constant pain. Dr. J. B. Lyman, of Rockford, was called as additional counsel. She now consented to the use of anæsthetics, and, accordingly, was put under the influence of chloroform. Dr. Lyman introduced a sound into the bladder, and, by digital examination, ascertained that there was some foreign substance in that viscus; and we came to the conclusion that it was a pessary. Upon further consultation, the patient was allowed to rest for the night, and Dr. Wm. H. Byford, of Chicago, was sent for, who, without much difficulty, effected its extraction.

There were present Drs. Lyman, DePuy, Mease, and myself. The patient was placed under the influence of chloroform, and another thorough examination made. The removal was accomplished by dilating the urthera, and extracting it with forceps. The little finger of the left hand was first passed into the bladder, and search made for the instrument, but it was beyond reach of this member. The forefinger of the left hand was next introduced, and the pessary could be felt so remote as to make it impossible to seize hold of it or exactly determine its position. The bladder contained several ounces of urine, and the instrument was easily displaced by the motion of the finger. After the urine was drawn off by a catheter, the index-finger was again inserted into the bladder, when, in consequence of the contraction of that organ, the pessary was brought down close to the pubis, at the opening into the urethra, and was easily controlled. By the forefinger, the pessary was drawn close up to the urethra, with the open end at the right hand of the operator, and the end of one of the branches placed as nearly as possible to the entrance of that canal. Ricord's phymosis forceps was next passed into the bladder, the end of the bar seized, and extraction commenced; but very soon the hold upon it gave way, and the instrument returned to its old position. It was again properly placed by the introducer's index-finger, and a second time taken hold of by the forceps. The bar was easily drawn out this time to the external orifices of the urethra, and by turning strongly down toward the vaginal opening, one-third of the instrument showed itself, and the balance quickly followed.

The time occupied by the efforts at extraction was about ten minutes. All present were surprised at the ease with which the fingers were passed through the urethra and the dexterity of the manœuvre that brought that disagreeable occupant of the bladder to light. As before remarked, the patient was a young married woman, and the genital organs were of virgin, etc., while the pessary was tolerably large. It measured one and three-quarters inches, across from one side to the other, and was two inches and a-half long. The diameter of the rod was three-eighths of an inch. The extracted instrument was incrusted with the urinary deposits pretty much all over. It had been in the bladder about three months. The patient promptly recovered, with no treatment, save the use of some light anodynes, and is progressing favorably in her pregnancy.

There can be no doubt, now, that this pessary was introduced into the bladder by mistake, instead of the vagina, which, I think, might very easily be effected. The physician, who was a man of experience, and had used the pessary frequently before, was sick, and laboring under the effects of opium, to such an extent as to be easily confused; and, in fact, though the pessary had disappeared, so that he could not find it, was entirely unable to account for its loss. Dr. H. R. Storer, of Boston, records the only other case, so far as I am informed, of this singular accident, and gives a very graphic description of the difficulties he met with when extracting it, in the *Medical Record*, of July 15th, 1868. Dr. H. W. Jones, of Chicago, a short time since, saw a case in which the pessary was accidentally introduced into the rectum, instead of the vagina.

